# A quarter of a century succession of epigaeic beetle assemblages in remnant habitats in an urbanized matrix (Coleoptera, Carabidae)

**DOI:** 10.3897/zookeys.147.1954

**Published:** 2011-11-16

**Authors:** Kamal J.K. Gandhi, Marc E. Epstein, Jessica J. Koehle, Foster F. Purrington

**Affiliations:** 1 Daniel B. Warnell School of Forestry and Natural Resources, The University of Georgia, Athens, Georgia, 30602, USA; 2 California Department of Food and Agriculture, Plant Pest Diagnostics Branch, 3294 Meadowview Road, Sacramento, California, 95832, USA; 3 City of Eagan, 3830 Pilot Knob Road, Eagan, Minnesota, 55122, USA; 4 The Ohio State University, Department of Entomology, Room 400 Aronoff Laboratories, 318 West 12th Avenue, Columbus, Ohio, 43201, USA

**Keywords:** Beetles, Carabidae, Cicindelinae, Coleoptera, Minnesota, remnant habitats, succession, urbanization

## Abstract

We studied the long-term (23–24 years) species turnover and succession of epigaeic beetle assemblages (Coleoptera: Carabidae, incl. Cicindelinae) in three remnant habitats [cottonwood (*Populus* spp.) and oak (*Quercus* spp.) stands, and old fields] that are embedded within highly urbanized areas in central Minnesota. A total of 9,710 beetle individuals belonging to 98 species were caught in three sampling years: 1980, 1981 and 2005 in pitfall traps in identical locations within each habitat. Results indicate that there were 2–3 times greater trap catches in 2005 than in 1980 (cottonwood and oak stands, and old fields) and 1.4–1.7 times greater species diversity of beetles in 2005 than in the 1980-1981 suggesting increased habitat association by beetles over time. Although there were no significant differences in catches between 2005 and 1981 (only cottonwood stands and old fields), there was a trend where more beetles were caught in 2005. At the species-level, 10 times more of an open-habitat carabid species, *Cyclotrachelus sodalis sodalis* LeConte, was caught in 2005 than in 1980. However, trap catches of five other abundant carabid species [*Pterostichus novus* Straneo, *Platynus decentis* (Say), *Platynus mutus* (Say), *Calathus gregarius* (Say), and *Poecilus lucublandus lucublandus* (Say)] did not change indicating population stability of some beetle species. These remnant habitats were increasingly colonized by exotic carabid species as *Carabus granulatus granulatus* Linneaus, *Clivina fossor* (Linneaus) and *Platynus melanarius* (Illiger), that were trapped for the first time in 2005. Species composition of epigaeic beetles was quite distinct in 2005 from 1980 with 39 species reported for the first time in 2005, indicating a high turnover of assemblages. At the habitat-level, greatest species diversity was in cottonwood stands and lowest was in old fields, and all habitat types in 2005 diverged from those in 1980s, but not cottonwood stands in 1981. As our sampled areas are among some of the last remnants of the original oak savanna habitats in central Minnesota, we hypothesize that conservation of these sites may be critical to maintaining epigaeic beetle assemblages under increased urbanization pressure.

## Introduction

Long-term forest succession deals with directional changes in communities (species abundance, diversity, and composition) within a specific physiographic context over time. Emphasis has been placed on understanding the rather early and contrasting changes in patterns and mechanisms of primary (e.g., during volcanic and glacial activity, and landslides), and secondary (e.g., following wild or prescribed fire) succession (Chapin et al. 1994). Post disturbance successional changes is well documented for plants (Bazzaz 1979; Denslow 1980), herbivorous insects (Torres 1992; Pascarella 1998), other animal species (Johnston and Odum 1956), and are also emerging for the more cryptic predatory insects (Holliday 1991; Work et al. 2004). However, less emphasis has been placed on forest succession occurring, perhaps more slowly, in mature forest stands and stable grassland landscapes, and this is especially true for remnants of native ecosystems in urban areas increasingly exposed to invasive and synanthropic species brought by urbanization and globalization.

Relatively undisturbed, undeveloped, green, or remnant areas embedded within major urban developments have become progressively rare and fragmented on the North American landscapes (Russell and Davis 2001). Although these remnant habitats may be influenced and stressed by the surrounding urbanization (McDonnell et al. 1997), they may enhance environmental quality (e.g., sequester carbon), and to some degree, preserve and maintain ecological processes within urban areas (Nowak and Crane 2002). Remnant habitats are considered to be crucial components of disturbed landscapes, as they provide refugia in which species negatively influenced by land use change may persist (Gandhi et al. 2001), and offer a network of islands and corridors of suitable habitat necessary for the maintenance of populations and communities characteristic of the native habitat (Noss 1987). In the future, these remnant habitats may serve as sources of biotic populations, and as benchmarks for habitat restoration activities of disturbed urbanized landscapes (Duelli et al. 1990), especially when long-term scientific data about the biotic and abiotic components of these ecosystems are available. Most importantly, these habitats that have remained relatively undisturbed and undeveloped may allow the persistence of late successional biotic assemblages within a landscape matrix of early seral stages maintained by frequent disturbances.

**Figure 1.**
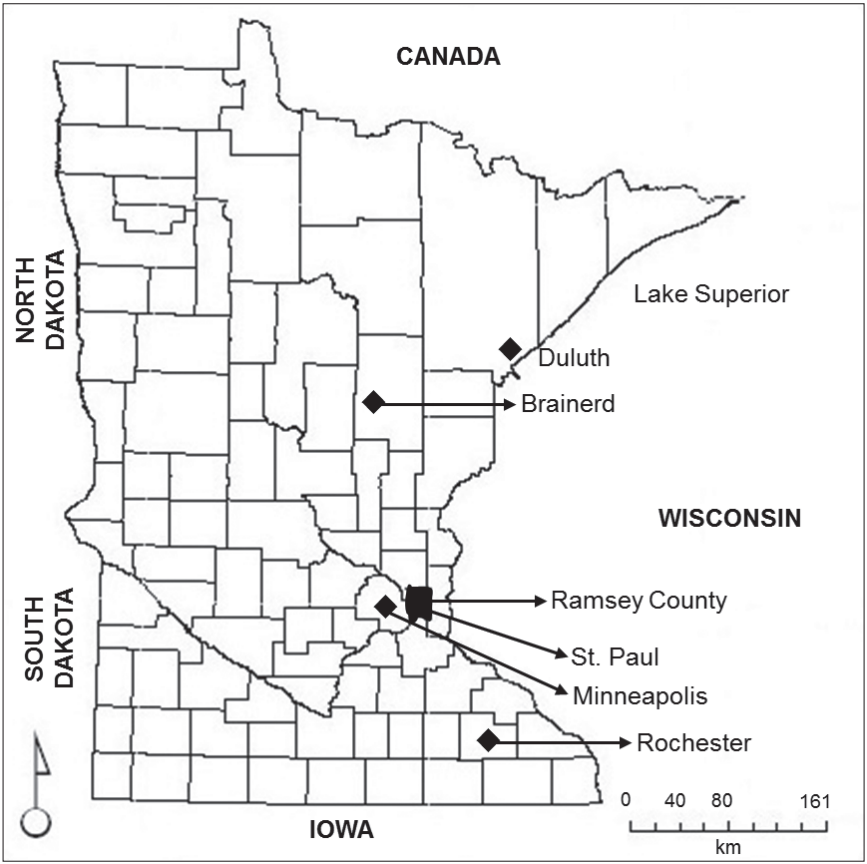
Location of study sites in Ramsey County and major cities in Minnesota, USA.

One of the few currently undeveloped areas around the highly urbanized areas are decommissioned army sites all over the United States. An excellent example is that of the Twin Cities Army Ammunition Plant (TCAAP) in Arden Hills Township in Ramsey County in Minnesota (Fig. 1). The TCAAP was originally built in 1941-1942 as an ammunition plant for World War II, and since that period, it has supported a variety of military and commercial uses (U.S. Department of Army 2001). Since the 1970s, TCAAP has been considered surplus by the Army, and environmental restoration and development of the area has been initiated during the past few years. At present, the TCAAP includes 931 ha, of which 486 ha is licensed to the U.S. National Guard, 46 ha is a part of undeveloped Rice Creek watershed, and 268 ha is being developed into residential and commercial property by Arden Hills Township (TCAAP 2005).

The TCAAP is considered a key link in one of the biggest ecological corridors north of the Twin Cities, running southwest from the Carlos Avery Wildlife Area to Rice Creek’s chain of lakes and into the Mississippi River (Embrace Open Space 2008). The habitats within TCAPP are diverse ranging from forests, wetlands, and grasslands as remnants of the original oak savanna habitat (Minnesota Department of Natural Resources 2005). Thus, they harbor rich communities of animal and plant species, and their communities have been the focus of several scientific studies over the past 30 years (Keenlyne 1976; Epstein 1982; Epstein and Kulman 1984, 1990; Minnesota Army National Guard 2001). During 1980-81, the co-author Epstein (then as a M.S. student at the University of Minnesota, Department of Entomology) intensively studied the habitat association patterns and phenology of ground beetle (Coleoptera: Carabidae) assemblages in the cottonwood (*Populus* spp.), oak (*Quercus* spp.), willow (*Salix* spp.), and field habitats in the TCAAP. From this study, Epstein (1982) documented sixty-six species of carabid beetles, and established a historical baseline for studies related to the succession of carabid assemblages in relatively undisturbed urban habitats. These habitats have remained largely undeveloped since the 1980s allowing natural succession characteristic of mature stands to occur.

The undeveloped habitats within TCAAP thus provided a unique opportunity to evaluate change of a largely predatory faunal community (carabid and tiger beetles) after a quarter of a century of natural succession processes in remnant ecosystems embedded in an urban matrix. During the summer of 2005, we re-sampled the habitats studied by Epstein (1982) in the TCAAP to better understand how the species abundance, diversity, and composition have changed over time within these relatively undisturbed habitats. Carabid and cicindelid beetles are ideal for this study as they are species-rich, abundant, easily sampled and identified, and are sensitive to changes in micro-habitat conditions rendering them first choice bioindicator taxa (Spence et al. 1997; Gandhi et al. 2008). Further, they are important predators in the forest soil, and are thus, hypothesized to be critical to forest processes such as nutrient cycling (Spence et al. 1996). Specifically, our research objectives were as follows: 1) to determine the mature successional changes of epigaeic beetle assemblages in remnant habitats within urbanized areas; and 2) to assess how successional changes in epigaeic beetles may vary as depending upon the habitat-type (cottonwoods, oaks, and old fields).

## Methods

### Study Sites

The forests in the TCAAP belongs to the Eastern broadleaf forest Province, Minnesota and southeast Iowa moraine Section, and St. Paul-Baldwin Plains and Moraines Sub-section (Minnesota Department of Natural Resources 1999) in the Ramsey County in central Minnesota (Fig. 1). The TCAAP contains a diversity of forested (oak and cottonwood), grassy (tall grass prairies), and riparian (Cattail marshes) areas. The soils belong to the orders Mollisols and Alfisols (Anderson et al. 2001), and are typically sandy-loamy in nature. In 1980, ten sites representing four habitats were selected according to dominant overstory plant species as follows: (1) northern pin oak (*Quercus ellipsoidalis* Hill) and white oak (*Quercus alba* L.) (four replicates termed as- NWO, HON, HOS, MLO); (2) eastern cottonwood (*Populus deltoides* Bartr. ex Marsh. var. *deltoides* ) and boxelder(*Acer negundo* L.) (three replicates- MCW, HCW, CWW); (3) black willow (*Salix nigra* Marsh) (one replicate- W); and (4) grasses in fields (two replicates- OFN and OFR). The grassy sites were primarily dominated by fescue (*Festuca* spp.) and Kentucky blue grass (*Poa pratensis* L.).

Although we did not conduct any formal vegetation inventories across the years, we noted some natural and anthropogenic changes in the sites in 2005 as follows: 1) MLO site had a greater abundance of *Prunus* spp. in the southern section; 2) HON site had an adjacent gravel pit that seemed to have become expanded, and the site was recently burned; 3) NWO site had a greater abundance of *Prunus* spp.; 4) MCW site had experienced some disturbance from vehicle tire tracks and tree removal; 5) CWW site was bordered by greater amount of standing water; 6) HCW site had an absence of downed trees, no longer had an understory of boxelder that was present in the 1980s, and was more open with *Rubus* spp. in the understory; and 7) in the W site, all the willows had died and the willow snags were standing in water.

### Beetle Sampling

In 1980, 1981, and 2005, epigaeic beetles were sampled using pitfall traps (Epstein 1982). The pitfall traps consisted of an outer 9.5 cm diameter by 12 cm high metal can without a bottom. A hole was dug in the ground, and the metal can was placed with upper edge flush on level with ground surface. A 16 ounce plastic cup was hung into the metal can and filled with 1-2 cm of propylene glycol (recreational vehicle antifreeze, Peak Co., Northbrook, Illinois) to retain fallen and preserve insects. A 64 cm^2^ plywood board was suspended 4 cm above the entire trap to minimize flooding and disturbances by small mammals. The pitfall traps were identical in design in all the years except in 1981 where three different pitfall trapping designs were used as follows: 1) traps with no aprons or a piece of board surrounding the top rim of the trap; 2) traps with aprons attached to the top rim of the trap; and 3) traps with aprons not attached to the top rim of the trap (*see* Epstein and Kulman 1984 for more details about these traps).

Pitfall traps were placed in identical stands and locations in all the three years to allow meaningful comparisons. In May 1980, traps were installed in locations chosen randomly from a grid (Epstein and Kulman 1990). Twenty traps were each installed in larger areas in NWO, MLO, HCW, OFN, and OFR, whereas 10 traps were each installed in HOS, HON, MCW, W, and CWW. In 1981, nine traps were each installed in OFN, OFR, HCW, MCW, and CWW along a linear transect. In May 2005, ten pitfall traps were each installed along a linear transect in the same locations used in the 1980s. The traps were spaced by 25 m to reduce inter-trap interactions, and placed > 25 m away from any habitat edges to reduce edge-effects. Pitfall traps were operated from May through September in 1981 and 2005, and June through September in 1980 (due to late snowfall), and were emptied every 10-15 days.

All adult carabid beetles including Cicindelinae (tiger beetles) were identified to species-level. The taxonomy of carabids follows that of Bousquet and Larochelle (1993) and Ball and Bousquet (2000). Representatives of the voucher specimens collected in 1980-1981 were borrowed from the University of Minnesota Insect Collection, and were re-identified to ensure consistency in species identifications across years. Voucher specimens collected in 2005 will be deposited at the University of Minnesota Insect Collection and the Minnesota Department of Natural Resources Collection.

### Statistical Analyses

Total trap catch data for all the years were standardized to 1,000 trap-days to account for trap disturbances and variable numbers of days the traps were operational across years. Analyses were conducted on a per-trap basis since the numbers of traps used were variable across years (Epstein 1982). Repeated measures analysis of variance tests (ANOVA) were used to detect differences in total trap catches across the years and habitat types (Zar 1996, SAS 2003). Since different sites were sampled in 1980 and 1981, we conducted following ANOVAs to compare trap catches between the following sites (replicates): 1) 1980 and 2005- cottonwood stands (MCW, HCW, and CWW), oak stands (NWO, MLO, HON, and HOS) and old fields (OFN and OFR); and 2) between 1981 and 2005- cottonwood stands (HCW, MCW, and CWW) and old fields (OFN and OFR). Although we sampled willow stands in 1980 and 2005, we removed this site from formal analyses due to only one replicate, and instead we qualitatively describe the changes in this site over years. Beetle numbers were transformed to a log-scale, after which they met the assumptions of normality and equal variance. Tukey-Kramer’s posthoc tests were used to assess differences within habitats. Similar analyses were performed for the six most abundant species (> 5% of the total catches) in our study.

Rarefaction indices were used to assess species diversity in 2-3 habitat types across three years (McCune and Mefford 1999; McCune and Grace 2002; Magurran 2004). Rarefaction is an especially useful technique to assess species diversity as it calculates mean species richness at the lowest sample size across all the habitat combinations, thus ensuring similar trapping effort. We created both rarefaction curves and determined the mean (+SE) species richness at the lowest subsample size within any habitat. Epigaeic beetle species compositions for year and habitat type were compared by constructing community-level dendrograms using standardized beetle catches per trap. Total beetle catches within each year and habitat combinations were analyzed by using the Bray-Curtis (or Sørenson) Distance analysis with the group average clustering option (McCune and Mefford 1999; McCune and Grace 2002). Both beetle species diversity and composition were compared across habitats in a similar way to species abundance: 1) between 1980 and 2005- cottonwood stands (MCW, HCW, and CWW), oak stands (NWO, MLO, HON, and HOS) and old fields (OFN and OFR); and 2) between 1981 and 2005- cottonwood stands (HCW, MCW, and CWW) and old fields (OFN and OFR).

## Results

Overall, a total of 9,710 beetle individuals belonging to 98 species were caught in 1980, 1981 and 2005 (Appendix I). During the summer of 1980, 1981, and 2005, we respectively caught 1,745; 1,850; and 6,105 beetles represented by 46, 43, and 86 species. *Cyclotrachelus sodalis sodalis* LeConte (1,594 individuals) was the most abundant beetle followed by *Pterostichus novus* Straneo (1,453), *Platynus decentis* (Say) (841), *Platynus mutus* (Say) (817), *Calathus gregarius* (Say) (599), and *Poecilus lucublandus lucublandus* (Say) (566). During our sampling in 2005, a total of 39 beetle species were found for the first time at the TCAAP, including new Minnesota state records for *Brachinus kavanaughi* Erwin, *Carabus granulatus granulatus* Linneaus, and *Trichotichnus autumnalis* (Say) (Appendix I). Along with *Carabus granulatus granulatus*, two other species new to TCAAP, *Clivina fossor* (Linneaus) and *Platynus melanarius* (Illiger), are exotic species from Europe (Bousquet and Larochelle 1993). Twenty-nine beetle species were shared between all three years of sampling (Appendix I).

**Figure 2.**
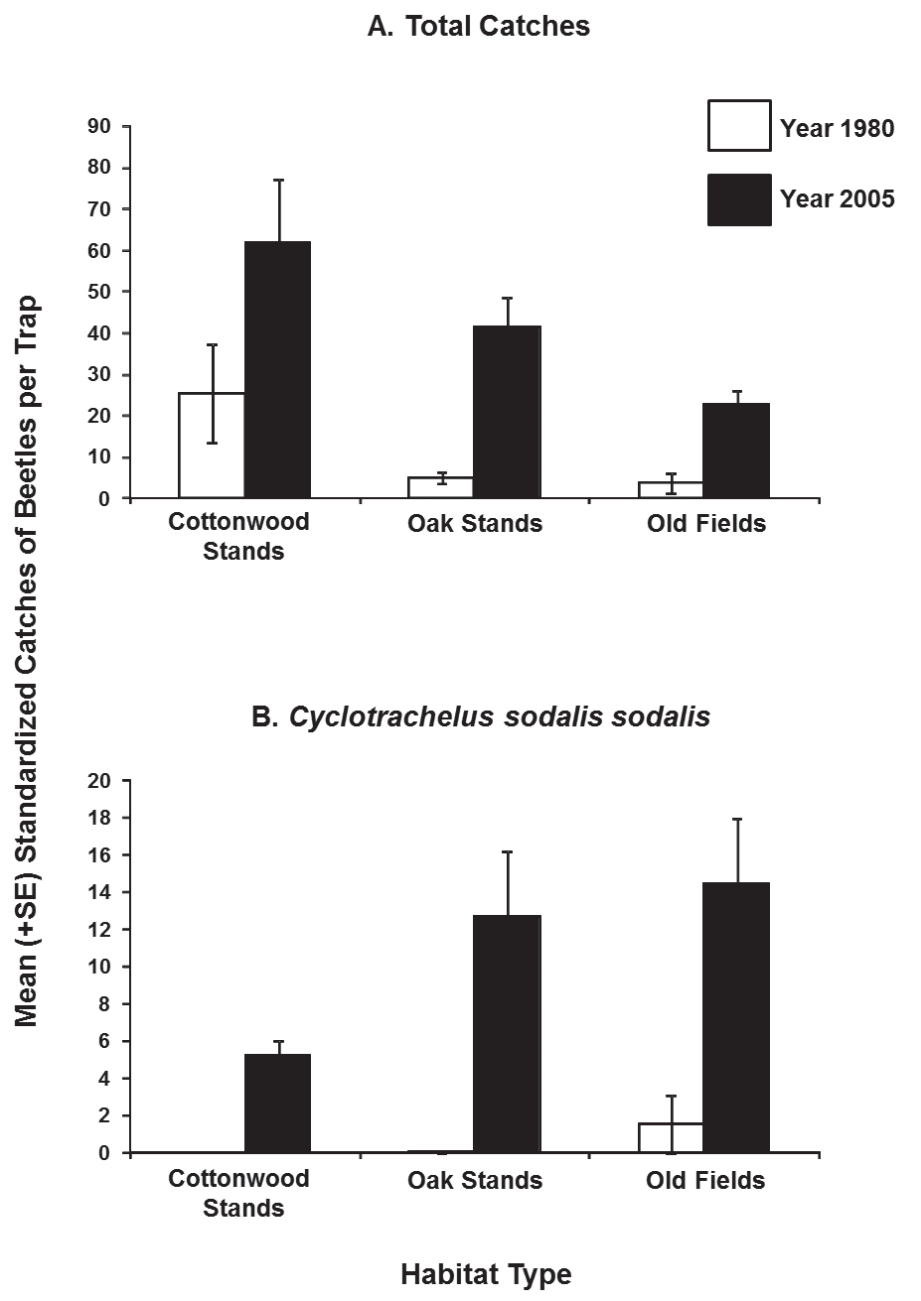
Mean (+SE) standardized total catches of epigaeic beetles (**A**), and *Cyclotrachelus sodalis sodalis* LeConte (**B**) caught in 1980 and 2005 in cottonwood (N = 3) and oak (N =4) stands, and old fields (N = 2).

For the total number of beetle catches for 1980 and 2005, there were significant differences between years (*F_1,6_* = 37.32; *P* < 0.001), but not between habitats (*F*_2,__6_ = 3.76; *P* = 0.087), or their interactions (*F_2,6_* = 1.29; *P* = 0.341). About 2-3 times more beetles were caught in 2005 than in 1980 across all habitats (Fig. 2A). For the total number of beetle catches for 1981 and 2005, there were no significant differences between years (*F_1,3_* = 4.02; *P* = 0.139), habitats (*F_2,6_* = 3.76; *P* = 0.087), or their interactions (*F_2,6_* = 0.01; *P* = 0.930). However, there was a trend where 1.5 times more beetles were caught in 2005 than in 1981. Since the interaction terms were not significant in either of the analyses, this suggests that the habitat associations of beetles had remained largely unchanged over time.

At species-level for 1980 and 2005, year (*F_1,6_* = 81.67; *P* < 0.001) and habitat type (*F_2,6_* = 5.35; *P* = 0.045) were significant factors for *Cyclotrachelus sodalis sodalis*. More than ten times the numbers of *Cyclotrachelus sodalis sodalis* individuals were caught in 2005 than in 1980 (Fig. 2B, Table 1). For *Poecilus lucublandus lucublandus* (*F_2,6_* = 4.96; *P* = 0.05) and *Pterostichus novus* (*F_2,6_* = 10.76; *P* = 0.01) habitat was a significant factor. At species-level for 1981 and 2005, habitat was also a significant factor for *Calathus gregarius* (*F_2,6_* = 9.82; *P* = 0.05). Tukey’s test failed to pick up specific differences among habitats for all the above four species, perhaps due to marginally significant *P*-value. There were trends where more individuals of *Cyclotrachelus sodalis sodalis* were caught in oak stands in 1980 and 2005, *Poecilus lucublandus lucublandus* and *Pterostichus novus* in cottonwood stands in 1980 and 2005, and *Calathus gregarius* in old fields in 1981 and 2005 (Table 1). Other species did not show a response to either years or habitat-types (*P* > 0.05).

**Table 1.** Mean (+ SE) trap catches of abundant carabid beetles in three habitats and sampling years.

**Beetle Species**	**Year of Sampling**	**Cottonwood(N = 3)**	**Oak(N = 4)**	**Old Field(N = 2)**
*Calathus gregarius*	1980	0.571 + 0.525	0.274 + 0.092	1.595 + 1.595
	1981	0.429 + 0.39	NA^†^	4.237 + 2.375
	2005	0.51 + 0.474	4.290 + 2.479	1.932 + 0.5
*Cyclotrachelus sodalis sodalis*	1980	0	0.048 + 0.048	1.545 + 1.545
	1981	0	NA^†^	2.212 + 2.214
	2005	5.226 + 0.835	12.704 + 3.523	14.42 + 3.56
*Platynus decentis*	1980	0.841 + 0.681	0.012 + 0.012	0
	1981	11.03 + 8.858	NA^†^	0
	2005	9.476 + 4.771	0.252 + 0.149	0
*Poecilus lucublandus lucublandus*	1980	0.889 + 0.229	0.381 + 0.381	0.667 + 0.619
	1981	0.778 + 0.387	NA^†^	1.369 + 0.886
	2005	8.856 + 6.361	0.361 + 0.338	0.332 + 0.332
*Pterostichus mutus*	1980	0.254 +.254	0.357 + 0.196	0
	1981	0.161 + 0.161	NA^†^	0
	2005	4.45 + 4.186	10.724 + 9.253	0.111 + 0.111
*Pterostichus novus*	1980	15.905 + 7.903	0.691 + 0.599	1.143 + 1.143
	1981	14.976 + 9.617	NA^†^	2.134 + 2.134
	2005	2.324 + 2.29	1.233 + 1.139	0.038 + 0.038

^**†**^NA- Not applicable as one of the habitats was not sampled in those years.

**Figure 3.**
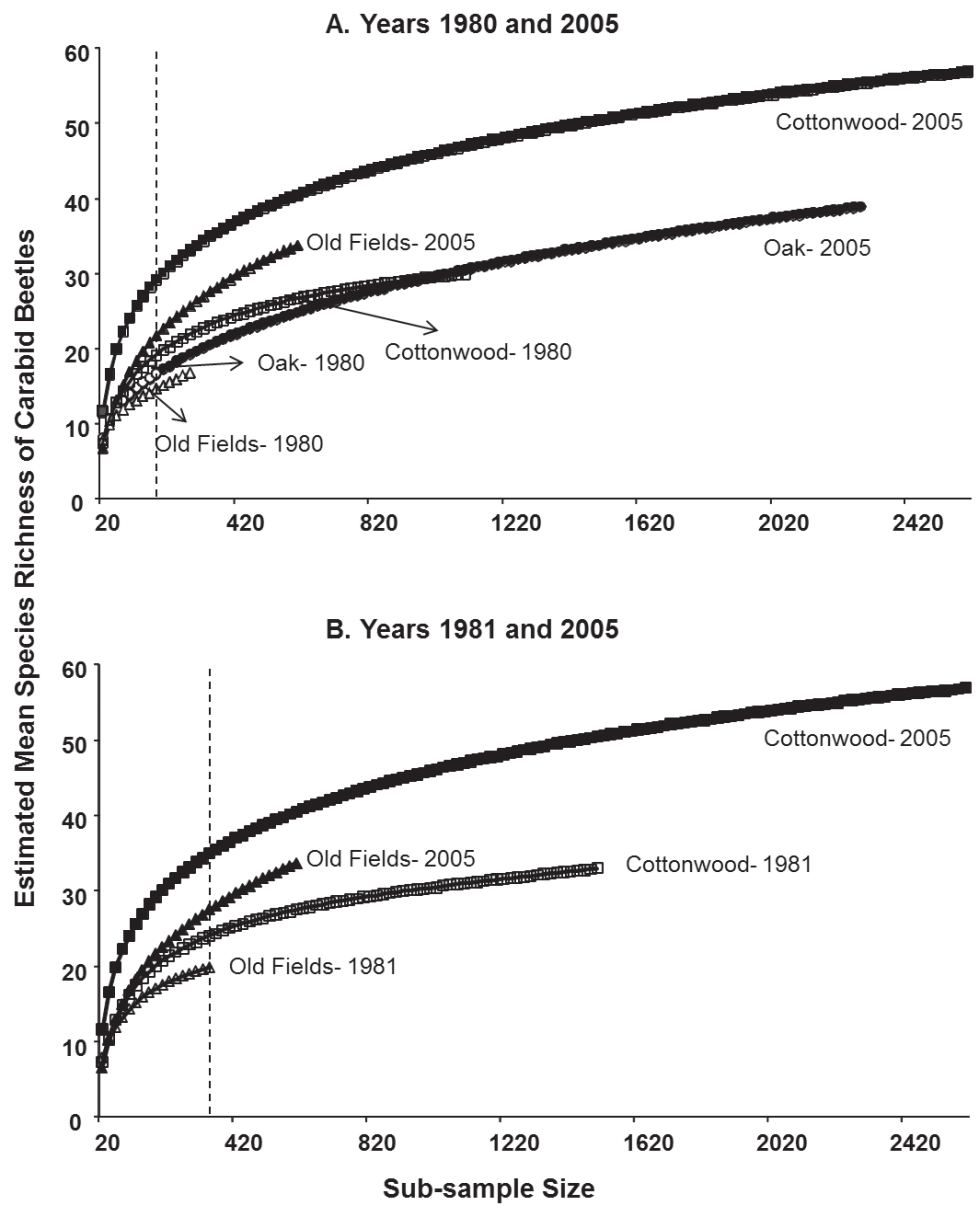
Estimated mean species richness of epigaeic beetles using rarefaction analyses in sampling years 1980 and 2005 (**A**) and 1981 and 2005 (**B**) in cottonwood and oak stands, and old fields.

Rarefaction results for 1980 and 2005 at the lowest subsample size of 180 individuals indicated that the cottonwood stands in both years had the highest species diversity followed by old fields in 2005, oaks stands in both years, and old fields in 1980 (Table 2, Fig. 3A). Similarly, rarefaction results for 1981 and 2005 at the lowest subsample size of 340 individuals also suggested that cottonwood stands in 2005 and old fields in 1981, respectively, had the highest and lowest species diversity (Table 2, Fig. 3B). In general, beetle species diversity increased about 1.4-1.7 times from 1980s to 2005 in cottonwood stands and old fields. Further, the species accumulation curve for cottonwood stands did not level out in our study, indicating that these habitats are quite diverse, and they can accommodate more species with a greater sub-sample size (Fig. 3).

**Table 2.** Mean (+ SE) estimated species richness of epigaeic beetles using rarefaction analyses for 1980, 1981, and 2005.

**Year of Sampling**	**Subsample Size**	**Cottonwood**	**Oak**	**Old Field**
1980	180	19.1 + 3.66	16.7 + 0.32	14.6 + 1.51
2005		29.2 + 5.59	16.6 + 3.88	21.7 + 4.6
1981	340	21.1 + 3.36	NA^†^	19.9 + 0.09
2005		35 + 5.97	NA^†^	27.6 + 3.82

^**†**^NA- Not applicable as one of the habitat was not sampled in those years.

**Figure 4.**
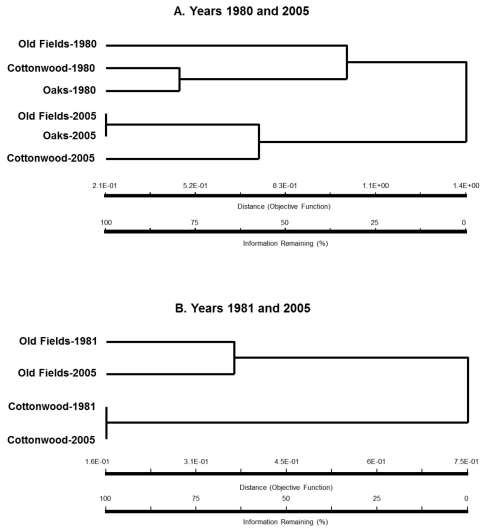
Dendrogram for the similarity/dissimilarity in standardized per trap catches of epigaeic beetle assemblages in sampling years 1980 and 2005 (**A**) and 1981 and 2005 (**B**) in cottonwood and oak stands, and old fields.

Dendrogram created using cluster analysis from standardized beetle catch data per trap for 1980 and 2005 revealed that the carabid beetle assemblages had diverged over time (Fig. 4A). Carabid beetle assemblages within all habitat-types in 1980 were quite dissimilar to that of 2005 (Fig. 4A). The old fields and oak stands were more similar to each other than to cottonwood stands in 2005. In contrast, dendrogram for years 1981 and 2005 revealed that the cottonwood stands had remained largely unchanged, however species composition of old fields in 1981 and 2005 were quite dissimilar to each other (Fig. 4B).

## Discussion

Overall, this study of remnant habitats in an urbanized matrix represents one of the few systematic and quantitative studies on arthropods where same habitats have been sampled over a long period of time, thus enabling a better understanding of natural succession. Further, our study illustrates the importance of using relatively undeveloped and surplused army areas for conducting long-term surveys and monitoring of arthropod populations and communities within urban areas. The five major successional trends evident in this study are as follows: 1) succession after a quarter century resulted in greater numbers (especially between 1980 and 2005), and species diversity of epigaeic beetles indicating greater habitat association by beetles; 2) some open-habitat species such as *Cyclotrachelus sodalis sodalis* became more common in 2005 than in 1980, whereas numbers of other native beetle species did not change; 3) these remnant habitats had an invasion of exotic carabid beetle species indicating a surrounding matrix effect of urbanization; 4) the species composition of epigaeic beetles was largely different after a quarter century suggesting a turnover of species; and 5) cottonwood forests in 2005, and old fields in 1980 and 1981, respectively had the greatest and lowest species diversity. We provide following mechanistic hypotheses for the above successional trends of epigaeic beetle assemblages in these habitats.

We caught significantly more beetles in 2005 than in 1980 in all the habitat types (cottonwood and oak stands, and old fields). In fact, in 2005, beetle trap catches increased 2-3 times as much than in 1980 in some sites indicating the increased importance of these habitats for carabid beetles. Although the results were not significant for catches between 2005 and 1981, we found similar trends of more beetles caught in 2005. Halme and Niemelä (1993), Ward et al. (2002), and Lemieux and Lindgren (2004) all noted that remnant woodland islands supported more individuals and/or species than the surrounding disturbed matrices characteristic of agricultural and forestry activities. The temporal patterns in our study may therefore, reflect an increase in urban development of the surrounding matrix, accentuating the role of our remnants as refugia for epigaeic beetle assemblages. One hypothesis to explain this trend is that epigaeic beetles may be immigrating to these remnant habitats from the surrounding urban matrix, especially those with fully developed wings. Conversely, it is also likely that the populations of beetles, especially those with reduced or fused hind-wings and thus, limited dispersal ability may be achieving greater reproductive success in the remnant habitats over time (Lindroth 1961-69; Den Boer 1970). There is some evidence for the latter hypothesis, as *Cyclotrachelus sodalis sodalis*, that was the most commonly caught beetle and has reduced hind-wings, was the only species that showed a significant change in trap-catches over 23 years. More *Cyclotrachelus sodalis sodalis* were caught each successive sampling year, with about 10 times more beetles caught in 2005 than in the 1980. *Cyclotrachelus sodalis sodalis* is typically found in open grassy areas (Epstein and Kulman 1990), and has also been collected near railroad tracks, pastures, and abandoned farmlands (Freitag 1969). Our results therefore, indicate that these habitats may have become more open, perhaps due to the ongoing small-scale gap dynamics and edge effects with positive effects on somewhat dispersal-limited and open-habitat native species.

Similar to trap catches, the species diversity of epigaeic beetles was 1.4-1.7 times greater in 2005 than in 1980 and 1981. Further, in 2005, we caught 39 beetle species including eight from the genus *Chlaenius* that had not been previously recorded in these remnant habitats. Some of these *Chlaenius* species are fully-winged and capable of flight, and this trend suggests invasion from elsewhere. Most of these beetle species were represented by only one or two individuals, and could be transients in these habitats. However, *Bembidion frontale* (LeConte) (total number of individuals = 98) and *Chlaenius impunctifrons* Say (173) that are hygrophilous species, and *Pterostichus melanarius* (226) that is an open-habitat and synanthropic species, were caught in sufficient numbers to indicate that they may have established reproductive populations in these habitats. Further, there is an apparent perplexing loss of 12 species in 2005, which were trapped in the 1980s. An obvious example is that of *Harpalus opacipennis* (Haldeman), which was previously relatively abundant in sandy soils in old fields, but was no longer found in 2005. Since, this species is mostly associated with open, dry areas with sandy soils (Lindroth 1961-69); its disappearance may indicate subtle changes in microhabitat conditions within old fields over time.

The numbers of exotic carabid beetles arriving and establishing in North America have increased dramatically within the past 30 years (Spence and Spence 1988; Gandhi, *unpublished data*). Further, these species have also increased their distribution range within the non-native habitat (Gandhi et al. 2005, 2008). For example, three exotic carabid species including *Carabus granulatus granulatus*, *Clivina fossor*, and *Pterostichus melanarius* were newly reported from these habitats in 2005, and *Carabus granulatus granulatus* is a new state record for Minnesota. All of these species are known to be synanthropic, and are associated with relatively open and disturbed habitats (Lindroth 1961-69; Spence and Spence 1988; Burke and Goulet 1998; Gandhi et al. 2005, 2008). It is therefore likely that these exotic species colonized these habitats from the surrounding urban areas suggesting an indirect effect of urbanization e.g., habitat changes and edges in these remnant habitats. Abundant numbers (226 individuals) of *Pterostichus melanarius*, an aggressive colonizer of disturbed habitats (Gandhi et al. 2008), were caught indicating that this beetle has established populations in these habitats. Further, *Pterostichus melanarius* was widely distributed in these habitats, as it was trapped in seven out of 10 sites. It is unknown whether the presence of these exotic carabid species, especially *Pterostichus melanarius*, may be problematic for the survival of native species such as its congener, *Pterostichus novus* in these habitats (Currie et al. 1996; Niemelä et al. 1997). Mechanistic studies are needed to determine if disappearance of certain species or weak trend of decreasing catches of *Pterostichus novus* in 2005 could be related to such an invasion.

Habitat association patterns of abundant epigaeic beetles at the stand-level were species-specific, as it has been documented in other studies from the boreal and sub-boreal forests to grasslands (Niemelä et al. 1992; Larsen et al. 2003; Pearce et al. 2003; Work et al. 2004; Gandhi et al. 2008), and that these patterns had largely remained unchanged over time. Although not significant, trends indicate that *Pterostichus novus* and *Poecilus lucublandus lucublandus* were caught in higher numbers in cottonwood stands, *Cyclotrachelus sodalis sodalis* in oak stands, and *Calathus gregarius* in old fields.Such habitat association results are not surprising, as *Pterostichus novus* is a forest species with records of being caught in moist hardwood stands, *Poecilus lucublandus lucublandus* is a generalist species with records in grassy habitats, and *Calathus gregarius* is typically present in open country and dry forests (Lindroth 1961-69). We also sampled willow stands in 1980 and 2005, however due to lack of replication (one stand only), we did not include willow in formal analyses. Similar to other stands, willow also showed a trend of increasing numbers of epigaeic beetles (4 times) in 2005 with twice the number of species (Appendix I). Most notably, *Cyclotrachelus sodalis sodalis* was caught for the first time in 2005 in these stands, probably reflecting habitat modifications.

There was a turnover of epigaeic beetle species (as depending upon habitat-types) from 1980 and 1981 to 2005, leading to quite different species composition over time. Twelve species of beetles that were present in the 1980 and 1981 were absent in 2005, and 39 species were recorded for the first time in 2005 (Appendix I). Similar results were found for other long-term studies such as the carabid fauna on Plummers Island (Maryland) where six species were not collected 11 years after first collection, and further, 11 species new to the site were recorded (Stork 1984). Purrington (1996) also documented a high faunal turnover of carabids on Nantucket Island (Massachusetts) from 1920s to 1995, as more than half the species in 2005 were not previously collected on the island. The beetle turnover within these habitats could be attributed either directly to low-level of disturbances present in these areas in 2005, and/or to natural succession occurring in the understory vegetation (which we did not document). Overlying these two factors could also have been changes in local weather patterns during 25 years under global climatic changes (Walther et al. 2002). According to the Wetlands Delineation Precipitation Data (WDPD) from year 1891 to 2005 at Shoreview (the nearest community to TCAAP), the intervening 25 years in our study had greater precipitation than ~25 years prior to the 1980s study (Minnesota Climatology Working Group 2009). Further, 2005 was in the 30^th^ percentile for the highest precipitation levels during the last 114 years of climate data collection (Minnesota Climatology Working Group 2009). In 2005, there was higher minimum summer temperature, greater snow-cover, and lower summer maximum temperature in our sites (G. Spoden, *personal communication*). These weather changes may have directly influenced ground beetles by altering their activity through longer growing periods and high precipitation levels in the summer as it has been found for other taxa (e.g., Crick et al. 1997). Conversely, weather changes may have indirectly affected epigaeic beetles through alterations in physical and chemical attributes of the soil-litter layer, and the abundance of prey and predator species in the soil-litter layer.

Some of our results in our study, especially when comparing 1981 and 2005, could be attributed to differences in pitfall trap designs, as slightly different designs were used in 1981 (with no aprons and with two kinds of aprons) and 2005 (with no aprons). Epstein and Kulman (1984) found that traps with no apron caught greater number of beetles than those with aprons. This reason could have led to the increased catches of beetles in 2005 than in 1981, however although there was such a trend, these results were not significant. Similarly, different types of traps may have caught different carabid beetle species in 1981 and 2005. However, we found that the species composition at least for cottonwood stands in 1981 and 2005 had remained largely unchanged over time.

## Conclusions

Succession of epigaeic beetles in these remnant habitats in an urban matrix indicates that there were greater trap catches, species diversity, and more distinct communities over 23-24 years. Further research is needed to assess whether these remnant islands in urban areas may differ from those present in forested landscapes (Gandhi et al. 2001, 2004), and whether remnant size, degree of isolation, and micro-habitat structure and composition are important factors in long-term maintenance of beetle assemblages. These remnant habitats are unfortunately, increasingly being invaded by exotic beetle species. The status of these habitats is further threatened under the TCAAP’s current proposed management plan (City of Arden Hills 2008), where a large portion of these areas are slated to be developed for residential purposes. We propose establishing these habitats as long-term monitoring areas, and providing them protection to ensure conservation and maintenance of these populations and communities of epigaeic beetles within the larger Twin Cities Metro area (McKinney 2002).

## Appendix I.

**Table:** Species-list of epigaeic beetles caught in 1980, 1981, and 2005 in four habitat-types.

**Carabid Beetle Species**	**Year 1980**				**Year 1981**			**Year 2005**			**Totals 1980**	**Totals 1981**	**Totals 2005**	**Total Catches**
	**Cottonwood**	**Oak**	**Old Field**	**Willow**	**Cottonwood**	**Old Field**	**Cottonwood**	**Oak**	**Old Field**	**Willow**				
*Acupalpus pumilus* Lindroth|	0	0	0	0	0	0	3	0	0	1	0	0	4	4
*Agonum gratiosum* (Mannerheim)¶	7	0	0	52	11	1	49	0	0	79	59	12	128	199
*Agonum melanarium* Dejean¶	6	0	0	5	41	0	290	2	0	37	11	41	329	381
*Agonum mutatum* (Gemminger and Harold)§	3	0	0	1	3	0	0	0	0	0	4	3	0	7
*Agonum palustre* Goulet§	0	0	0	11	0	0	0	0	0	0	11	0	0	11
*Agonum placidum* (Say)	0	1	0	0	0	0	0	1	0	0	1	0	1	2
*Agonum retractum* LeConte	0	0	0	0	1	0	4	1	0	6	0	1	11	12
*Agonum sordens* Kirby§	0	0	0	1	0	0	0	0	0	0	1	0	0	1
*Amara angustata* (Say)	0	0	1	0	0	0	0	0	7	0	1	0	7	8
*Amara apricaria* (Paykull)§	1	0	0	0	0	0	0	0	0	0	1	0	0	1
*Amara cupreolata* Putzeys¶	13	5	0	23	30	0	51	6	7	68	41	30	132	203
*Amara impuncticollis* (Say)|	0	0	0	0	0	0	0	1	0	0	0	0	1	1
*Amara laevipennis* Kirby§	2	0	0	19	5	1	0	0	0	0	21	6	0	27
*Amara latior* (Kirby)	0	1	0	0	0	0	0	3	0	0	1	0	3	4
*Amara obesa* (Say) §	0	0	10	0	0	0	0	0	0	0	10	0	0	10
*Anisodactylus harrisii* LeConte	0	0	0	1	0	0	9	0	3	1	1	0	13	14
*Anisodactylus kirbyi* Lindroth|	0	0	0	0	0	0	9	1	0	6	0	0	16	16
*Anisodactylus merula* (Germar)¶	0	0	1	0	0	1	1	0	1	0	1	1	2	4
*Anisodactylus nigerrimus* (Dejean)	0	0	0	0	1	0	3	0	10	0	0	1	13	14
*Anisodactylus rusticus* (Say)¶	0	0	1	0	0	1	0	0	14	0	1	1	14	16
*Anisodactylus verticalis* (LeConte)|	0	0	0	0	0	0	0	1	0	0	0	0	1	1
*Badister notatus* Haldeman	0	0	0	0	0	2	4	2	1	0	0	2	7	9
*Badister obtusus* LeConte¶	1	2	0	0	1	0	3	0	0	0	3	1	3	7
*Badister parviceps* Ball|	0	0	0	0	0	0	1	0	0	0	0	0	1	1
*Badister transversus* Casey†, §	0	0	0	0	1	0	0	0	0	0	0	1	0	1
*Bembidion frontale* (LeConte)|	0	0	0	0	0	0	98	0	0	0	0	0	98	98
*Bembidion graciliforme* Hayword	0	0	0	0	6	0	42	0	0	0	0	6	42	48
*Bembidion nigrivestis* Bousquet|	0	0	0	0	0	0	17	0	0	0	0	0	17	17
*Bembidion practicola* Lindroth	0	0	0	0	2	0	1	0	0	0	0	2	1	3
*Bembidion pseudocatum* Lindroth	0	0	0	0	3	0	8	0	0	0	0	3	8	11
*Bembidion quadrimaculatum oppositum* Say|	0	0	0	0	0	0	20	1	0	0	0	0	21	21
*Brachinus kavanaughi* Erwin†, |	0	0	0	0	0	0	1	0	0	0	0	0	1	1
*Bradycellus badipennis* (Haldeman)†, |	0	0	0	0	0	0	1	0	0	0	0	0	1	1
*Bradycellus lecontei* Csiki|	0	0	0	0	0	0	1	0	0	0	0	0	1	1
*Bradycellus lugubris* (LeConte) ¶	0	0	0	3	1	0	4	0	0	0	3	1	4	8
*Calathus gregarius* (Say) ¶	35	18	76	0	16	105	21	274	52	2	129	121	349	599
*Calleida punctata* LeConte|	0	0	0	0	0	0	0	0	0	1	0	0	1	1
*Calosoma calidum* (Fabricius)§	0	0	0	0	1	9	0	0	0	0	0	10	0	10
*Calosoma frigidum* Kirby¶	3	1	0	0	9	0	1	1	0	0	4	9	2	15
*Carabus granulatus granulatus* Linneaus †, ‡, |	0	0	0	0	0	0	1	0	0	0	0	0	1	1
*Carabus maeander* Fischer von Waldheim|	0	0	0	0	0	0	0	0	0	6	0	0	6	6
*Carabus serratus* Say	1	12	0	0	0	0	10	131	2	0	13	0	143	156
*Chlaenius emarginatus* Say|	0	0	0	0	0	0	0	1	0	0	0	0	1	1
*Chlaenius impunctifrons* Say|	0	0	0	0	0	0	165	4	0	4	0	0	173	173
*Chlaenius lithophilus* Say|	0	0	0	0	0	0	1	0	0	0	0	0	1	1
*Chlaenius nemoralis* Say|	0	0	0	0	0	0	0	1	0	0	0	0	1	1
*Chlaenius niger* Randall|	0	0	0	0	0	0	1	0	0	0	0	0	1	1
*Chlaenius pennsylvanicus pennsylvanicus* Say|	0	0	0	0	0	0	0	2	0	1	0	0	3	3
*Chlaenius purpuricollis purpuricollis* Randall|	0	0	0	0	0	0	0	0	1	0	0	0	1	1
*Chlaenius sericeus sericeus* (Forster)|	0	0	0	0	0	0	3	0	3	0	0	0	6	6
*Chlaenius tomentosus tomentosus* (Say)§	0	0	0	0	0	2	0	0	0	0	0	2	0	2
*Cicindela sexguttata* Fabricius	0	0	0	0	0	0	12	5	11	3	0	0	31	31
*Cicindela punctulata punctulata* Olivier|	0	0	0	0	0	0	0	0	1	0	0	0	1	1
*Clivina fossor* (Linnaeus)‡, |	0	0	0	0	0	0	9	0	1	14	0	0	24	24
*Cyclotrachelus sodalis sodalis* (LeConte)¶	4	0	65	0	0	55	213	701	388	168	69	55	1470	1594
*Cymindis americanus* Dejean¶	44	11	1	0	25	7	45	101	14	0	56	32	160	248
*Cymindis cribricollis* Dejean§	4	0	0	0	0	0	0	0	0	0	4	0	0	4
*Cymindis pilosus* Say¶	0	0	1	0	0	6	0	0	7	0	1	6	7	14
*Cymindis platicollis* (Say)†, |	0	0	0	0	0	0	0	1	0	0	0	0	1	1
*Cyminidis neglectus* Haldeman	0	0	0	0	0	3	0	3	9	0	0	3	12	15
*Dicaelus sculptilus upioides* Ball¶	142	55	15	0	57	8	37	89	11	2	212	65	139	416
*Diplocheila impressicollis* (Dejean)|	0	0	0	0	0	0	1	0	0	0	0	0	1	1
*Diplocheila obtusa* (LeConte)	0	0	0	0	0	3	0	0	2	0	0	3	2	5
*Diplocheila striatopunctata* (LeConte)|	0	0	0	0	0	0	2	0	0	1	0	0	3	3
*Diplocheila undulata* Carr|	0	0	0	0	0	0	2	0	0	0	0	0	2	2
*Harpalus caliginosus* (Fabricius)|	0	0	0	0	0	0	0	1	0	0	0	0	1	1
*Harpalus compar* LeConte¶	3	1	6	0	1	0	0	4	0	0	10	1	4	15
*Harpalus faunus* Say	0	0	1	0	0	0	0	0	1	0	1	0	1	2
*Harpalus herbivagus* Say|	0	0	0	0	0	0	0	0	1	0	0	0	1	1
*Harpalus opacipennis* (Haldeman)§	0	0	23	0	0	35	0	0	0	0	23	35	0	58
*Harpalus providens* Casey¶	4	1	0	0	0	0	2	18	0	0	5	0	20	25
*Harpalus somnulentus* Dejean¶	2	2	0	1	5	3	19	14	2	2	5	8	37	50
*Helluomorphoides praeustus bicolor* (T.W. Harris)§	0	0	2	0	0	0	0	0	0	0	2	0	0	2
*Lachnocrepis parallela* (Say)|	0	0	0	0	0	0	0	0	0	1	0	0	1	1
*Loricera pilicornis pilicornis* (Fabricius)|	0	0	0	0	0	0	0	0	1	0	0	0	1	1
*Oodes fluvialis* LeConte|	0	0	0	0	0	0	0	0	0	1	0	0	1	1
*Oxypselaphus pusillus* (LeConte)¶	9	0	0	2	17	0	102	16	0	42	11	17	160	188
*Pasimachus elongatus* LeConte¶	0	0	16	0	0	22	0	0	31	0	16	22	31	69
*Patrobus longicornis* (Say)	0	0	0	0	5	0	2	0	0	0	0	5	2	7
*Platynus decentis* (Say)¶	31	0	0	4	411	0	381	14	0	0	35	411	395	841
*Poecilus lucublandus lucublandus* (Say)¶	34	16	28	6	29	34	363	20	9	27	84	63	419	566
*Pterostichus caudicallis* (Say)¶	15	0	0	2	44	0	54	3	0	1	17	44	58	119
*Pterostichus commutabilis* (Motschulsky)|	0	0	0	0	0	0	2	0	0	10	0	0	12	12
*Pterostichus corvinus* (Dejean)¶	17	0	0	0	4	0	3	0	1	4	17	4	8	29
*Pterostichus femoralis* (Kirby)¶	2	0	0	0	15	0	12	1	2	0	2	15	15	32
*Pterostichus luctuosus* (Dejean)¶	7	0	0	1	20	0	59	0	0	7	8	20	66	94
*Pterostichus melanarius* (Illiger)‡, |	0	0	0	0	0	0	78	130	16	2	0	0	226	226
*Pterostichus mutus* (Say)¶	18	14	0	0	6	0	183	593	3	0	32	6	779	817
*Pterostichus novus* Straneo¶	562	3	48	12	558	53	96	69	1	51	625	611	217	1453
*Pterostichus patruelis* (Dejean)|	0	0	0	0	0	0	0	0	0	6	0	0	6	6
*Pterostichus pensylvanicus* LeConte¶	27	33	0	0	137	0	64	6	0	0	60	137	70	267
*Pterostichus permundus* (Say)|	0	0	0	0	0	0	0	0	1	0	0	0	1	1
*Selenophorus opalinus* (LeConte)	0	0	1	0	0	0	1	0	2	0	1	0	3	4
*Stenolophus conjunctus* (Say)	1	0	0	0	0	0	8	2	2	0	1	0	12	13
*Synuchus impunctatus* (Say)¶	31	92	7	0	32	3	37	74	0	14	130	35	125	290
*Trechus apicalis* Motschulsky¶	1	0	0	0	8	0	7	0	0	0	1	8	7	16
*Trichotichnus autumnalis* (Say)†, |	0	0	0	0	0	0	0	1	0	0	0	0	1	1
*Xestonotus lugubris* (Dejean)|	0	0	0	0	0	0	0	0	0	3	0	0	3	3
**Total Number of Beetles**	**1030**	**268**	**303**	**144**	**1506**	**354**	**2617**	**2299**	**618**	**571**	**1745**	**1860**	**6105**	**9710**
**Total Number of Species**	**30**	**17**	**18**	**16**	**33**	**20**	**57**	**39**	**34**	**31**	**46**	**43**	**86**	**98**

† New species records for Minnesota

‡ Introduced Species

§ Caught only in 1980-1981

| Caught only in 2005

¶ Found in all three years of study
